# Brainstem Organoids From Human Pluripotent Stem Cells

**DOI:** 10.3389/fnins.2020.00538

**Published:** 2020-06-26

**Authors:** Nobuyuki Eura, Takeshi K. Matsui, Joachim Luginbühl, Masaya Matsubayashi, Hitoki Nanaura, Tomo Shiota, Kaoru Kinugawa, Naohiko Iguchi, Takao Kiriyama, Canbin Zheng, Tsukasa Kouno, Yan Jun Lan, Pornparn Kongpracha, Pattama Wiriyasermkul, Yoshihiko M. Sakaguchi, Riko Nagata, Tomoya Komeda, Naritaka Morikawa, Fumika Kitayoshi, Miyong Jong, Shinko Kobashigawa, Mari Nakanishi, Masatoshi Hasegawa, Yasuhiko Saito, Takashi Shiromizu, Yuhei Nishimura, Takahiko Kasai, Maiko Takeda, Hiroshi Kobayashi, Yusuke Inagaki, Yasuhito Tanaka, Manabu Makinodan, Toshifumi Kishimoto, Hiroki Kuniyasu, Shushi Nagamori, Alysson R. Muotri, Jay W. Shin, Kazuma Sugie, Eiichiro Mori

**Affiliations:** ^1^Department of Neurology, Nara Medical University, Kashihara, Japan; ^2^Department of Future Basic Medicine, Nara Medical University, Kashihara, Japan; ^3^Laboratory for Advanced Genomics Circuit, RIKEN Center for Integrative Medical Sciences, Yokohama,Japan; ^4^Department of Molecular Biology, University of Texas Southwestern Medical Center, Dallas, TX, United States; ^5^Laboratory of Biomolecular Dynamics, Department of Collaborative Research, Nara Medical University, Kashihara, Japan; ^6^Department of Radiation Oncology, Nara Medical University, Kashihara, Japan; ^7^Department of Neurophysiology, Nara Medical University, Kashihara, Japan; ^8^Department of Integrative Pharmacology, Graduate School of Medicine, Mie University, Tsu, Japan; ^9^Department of Laboratory Medicine and Pathology, National Hospital Organization Kinki-Chuo Chest Medical Center, Sakai, Japan; ^10^Department of Obstetrics and Gynecology, Nara Medical University, Kashihara, Japan; ^11^Department of Orthopaedic Surgery, Nara Medical University, Kashihara, Japan; ^12^Department of Psychiatry, Nara Medical University, Kashihara, Japan; ^13^Department of Molecular Pathology, Nara Medical University, Kashihara, Japan; ^14^Department of Pediatrics, University of California, San Diego, San Diego, CA, United States; ^15^Department of Cellular and Molecular Medicine, University of California, San Diego, San Diego, CA, United States

**Keywords:** brain organoids, brainstem, neural crest, midbrain, dopaminergic neurons, human pluripotent stem cells, melanocyte

## Abstract

The brainstem is a posterior region of the brain, composed of three parts, midbrain, pons, and medulla oblongata. It is critical in controlling heartbeat, blood pressure, and respiration, all of which are life-sustaining functions, and therefore, damages to or disorders of the brainstem can be lethal. Brain organoids derived from human pluripotent stem cells (hPSCs) recapitulate the course of human brain development and are expected to be useful for medical research on central nervous system disorders. However, existing organoid models are limited in the extent hPSCs recapitulate human brain development and hence are not able to fully elucidate the diseases affecting various components of the brain such as brainstem. Here, we developed a method to generate human brainstem organoids (hBSOs), containing midbrain/hindbrain progenitors, noradrenergic and cholinergic neurons, dopaminergic neurons, and neural crest lineage cells. Single-cell RNA sequence (scRNA-seq) analysis, together with evidence from proteomics and electrophysiology, revealed that the cellular population in these organoids was similar to that of the human brainstem, which raises the possibility of making use of hBSOs in investigating central nervous system disorders affecting brainstem and in efficient drug screenings.

## Introduction

The brainstem is a posterior region of the brain between the deep structures of the cerebral hemispheres. It connects the cerebrum with the spinal cord and is divided into three parts: midbrain, pons, and medulla oblongata. They contain multiple nuclei and small fiber tracts widely projecting to the cerebrum cortex, basal ganglia, and other parts of the cerebrum. Brainstem functions such as alertness, heartbeat, blood pressure, and respiration are considered to be more vital for life than that of the cortex. Therefore, damages to or disorders of brainstem including infarction, hemorrhage, tumors, or any neurodegenerative diseases may lead to death. To investigate the pathology of these diseases and to establish novel therapies, models recapitulating brainstem tissue are needed.

Recent progress on protocols for inducing organs in-a-dish (organoids) provides potentials for the modeling of various diseases (Clevers, 2016). Organoids mimic the structure of organs composed of various cells such as the kidney (Takasato et al., 2015), brain (Dang et al., 2016), colon (Sato et al., 2009), and retina (Eiraku et al., 2011; Nakano et al., 2012). The use of brain organoids is a recognized method for the recapitulation of human fetal development during *in vitro* cultivation (Lancaster et al., 2013; Lancaster and Knoblich, 2014; Trujillo et al., 2019).

However, improvements to the protocols are still needed, particularly in aspects such as the maturity, efficiency, and the extent of recapitulation captured in the organoids. Recently, a protocol for generating human midbrain-like organoids from human pluripotent stem cells (hPSCs) was reported (Jo et al., 2016). There are also reports on the effects of reagents or growth factors on the differentiation of dopaminergic neurons (Diaz et al., 2009; Ayton et al., 2016; Lee et al., 2016). Based on these findings, we designed a new method for generating a human brainstem organoid (hBSO) model where the midbrain, surrounding brainstem parts, and neural crest region behind them are induced by the addition of basic fibroblast growth factor (bFGF) and epidermal growth factor (EGF) for neuronal stem/progenitor cells expansion. This is followed by treatment with brain-derived neurotrophic factor (BDNF), glial cell line–derived neurotrophic factor (GDNF), neurotrophin 3 (NT-3), cyclic adenosine monophosphate (cAMP), and ascorbic acid for the differentiation of dopaminergic neurons. In the present study, we established a novel method for inducing hBSOs. We believe our methods will become a powerful tool in examining the pathology of neurodegenerative or neurodevelopmental diseases affecting the brainstem.

## Materials and Methods

### Cell Culture

Human induced pluripotent stem cells (iPSCs) and embryonic stem cells (ESCs) are maintained in feeder-free condition with mTeSR1 media. Human iPSC line (XY) was obtained from Takara, Kusatsu, Shiga, Japan, and human H9 ESC line (WA09) was purchased from WiCell Research Institute, Madison, WI, United States. Embryonic stem cells and iPSCs were cultivated in mTeSR1 medium (Stemcell Technologies, Vancouver, British Columbia, Canada), based on feeder-free culture protocols on six-well plates (Corning, Corning, NY, United States), coated with growth factors reduced Matrigel (BD Biosciences, San Jose, CA, United States). At the time of passage, we added ROCK inhibitor (final concentration 10 μM; Selleck Chemicals, Houston, Texas, United States). These cells were maintained with daily medium change without ROCK inhibitor until they reached approximately 70% confluence. Then, they were detached by Versene Solution (Thermo Fisher Scientific, Waltham, MA, United States) and seeded by 1:20 dilution ratio.

### Human Brainstem Organoid Generation

The hBSOs were generated with some modifications on the cerebral cortical organoid protocol (Thomas et al., 2017; Trujillo et al., 2018). Human iPSCs/ESCs were gently dissociated by 10 min of treatment with 50% Accutase (Sigma A6964) in phosphate-buffered saline (PBS). Detached cells were transferred to six-well plates at the density of four million cells in 5 ml mTeSR1 medium with 5 μM ROCK inhibitor, 1 mM dorsomorphin (Wako, 040-33753) and 10 μM SB431542 (Cayman Chemical, 13031) per well in six-well plates on the orbit shaker (WakenBtech) to keep the cells in suspension. For neural induction from day 3, media was switched to one composed of neurobasal medium (Thermo Fisher Scientific, Waltham, MA, United States) and 2× Gem21NeuroPlex (Gemini Bio-Products, CA, United States), 1× non-essential amino acid solution (NEAA, Sigma-Aldrich), 1× GlutaMAX (Thermo Fisher Scientific, Waltham, MA, United States), 1 mM dorsomorphin, 10 μM SB431542, 10 μM transferrin, 5 mg/L human insulin, and 0.063 mg/L progesterone. After 9 days of exposure to dorsomorphin and SB431542, we treated the cells with 20 ng/mL bFGF (Peprotech, AF-100-18B) to induce neural progenitor cell (NPC) proliferation in the presence of neurobasal-A medium (Thermo Fisher Scientific, Waltham, MA, United States), supplemented with 2× Gem21NeuroPlex, 1× NEAA, 1× GlutaMax, 10 μM transferrin, 5 mg/L human insulin, and 0.063 mg/L progesterone until day 16. Cells were then kept in the same media containing not only 20 ng/mL bFGF, but also 20 ng/mL EGF (Wako, 059-07873) until day 22. After day 22, EGF and bFGF were replaced by ascorbic acid (nacalai, 13048-42), cAMP (nacalai, 11540-74), BDNF (Wako, 028-16451), GDNF (Wako, 075-04153), and NT-3 (Peprotech, 450-03). After day 28, cells were cultivated without any growth factors for neuronal maturation. Organoid results were combined from at least three separate batches of inductions.

### Human Cerebral Organoid Generation

The human cerebral organoids (hCOs) were generated as per previously reported protocols (Lancaster et al., 2013; Lancaster and Knoblich, 2014). Human iPSCs/ESCs were detached and subjected to embryoid body (EB) induction using the protocol. After 4 days, half of the media was replaced by human EB medium without ROCK inhibitor and bFGF. After 2 days, the EBs were transferred into a neural induction media and embedded in Matrigel after 5 days. The organoids were subsequently induced by the use of an orbital shaker, following the original protocol.

### Immunohistochemical Analysis

Each human cerebral or brainstem organoid was fixed in 4% paraformaldehyde in PBS overnight at 4°C, dehydrated with 30% sucrose in PBS and embedded in O.C.T. compound (Thermo Fisher Scientific, Waltham, MA, United States). Cryostat sections (14 μm) were cut and mounted onto slides (Thermo Fisher Scientific, Waltham, MA, United States). Mounted sections were incubated for 1 h at room temperature with blocking solution [3% normal goat serum + 0.3% Triton X-100 in Tris-buffered saline (TBS)] and incubated with primary antibodies (Supplementary Table S1) diluted in blocking solution overnight at 4°C. After three washes with TBS, corresponding fluorophore-conjugated secondary antibodies diluted in the blocking solution were added and incubated for 2 h at room temperature and followed by DAPI staining. Finally, stained slides were rinsed with TBS three times, mounted, and analyzed using a FV3000 Confocal Microscope (Olympus, Shinjuku, Tokyo, Japan).

### RNA Isolation, Reverse Transcriptase–Polymerase Chain Reaction, and Quantitative Polymerase Chain Reaction

RNA from hCOs/hBSOs and ESCs was extracted according to the protocol supplied with TRIzol reagent (15596018; Thermo Fisher Scientific, Waltham, MA, United States). The concentration and purity of the RNA samples were measured using Spectrophotometer (Beckman Coulter, Brea, CA, United States). Extracted RNA samples were either shipped to bioengineering laboratory for RNA sequencing analysis or subjected to reverse transcriptase–polymerase chain reaction (RT-PCR). For RT-PCR, the extracted RNAs were reverse transcribed according to the protocol supplied with ReverTra Ace qPCR RT Master Mix (FSQ-201; TOYOBO, Osaka, Osaka, Japan). StepOne Plus Real-time PCR System (Thermo Fisher Scientific, Waltham, MA, United States) was used to amplify and quantify levels of target gene cDNA. Real-time quantitative RT-PCR (qRT-PCR) was performed with SsoAdvanced Universal SYBR Green Supermix (172-5271; Bio-Rad Laboratories, Hercules, CA, United States) and specific primers for qRT-PCR (Supplementary Table S2). The cycling conditions for PCR program were 2 min at 95°C for activation followed by 40 cycles of 95°C, over a duration of 5 s for denaturation, 60°C for 30 s for annealing, 95°C for 15 s, 60°C for 30 s, and 95°C for 15 s for melt curve stage. Reactions were run in triplicate. The expression of each gene was normalized to the geometric mean of β-actin as a housekeeping gene and analyzed using the ΔΔCT method. Mean threshold cycle values of each gene in qPCR are shown in Supplementary Tables S3 and S4. Statistical significance was calculated by a two-tailed Student *t* test. A *p* value of less than 0.05 was considered statistically significant.

### RNA Sequencing

Total RNA was isolated from cells using the PureLink RNA Mini Kit (12183018A) according to the manufacturer’s instructions. RNA concentration was analyzed by Qubit RNA HS Assay Kit (Thermo Fisher Scientific, Waltham, MA, United States), and the purity was assessed using the Qsep100 DNA Fragment Analyzer and RNA R1 Cartridge (BiOptic, New Taipei City, Taiwan). Subsequently, total RNA was converted to cDNA and used for Illumina sequencing library preparation based on the KAPA Stranded mRNA-Seq Kit protocols (KAPA Biosystems, Wilmington, MA, United States). DNA fragments were then subjected to adapter ligation, where dsDNA adapters with 3’-dTMP overhangs were ligated to A-tailed library insert fragments by FastGene Adapter Kit (NIPPON Genetics, Bunkyo, Tokyo, Japan). The purified cDNA library products were evaluated using Qubit and dsDNA HS Assay Kit (Thermo Fisher Scientific, Waltham, MA, United States), followed by quality assessment using the Fragment Analyzer and dsDNA 915 Reagent Kit (Advanced Analytical Technologies, Ankeny, IA, United States) and finally by sequencing (2 × 75 bp) on NextSeq 500 (Illumina, San Diego, CA, United States).

### Transcriptome Analysis

A count-based differential expression analysis “TCC” was used to identify differently expressed genes (DEGs) in the RNA-seq data with a thresholded false discovery rate of 20% (Sun et al., 2013). iRegulon (Janky et al., 2014) was used to identify transcription factors (TFs) potentially regulating the DEG with normalized enrichment scores >4 as the threshold. Genotype-Tissue Expression (GTEx) (GTEx Consortium, 2013) was used to analyze the similarity of expression pattern between organoids and various tissues in the brain.

### Electrophysiology

Electrophysiological recordings of the cells in hBSOs at 3 months were performed. An organoid was transferred to a glass-bottom recording chamber on an upright microscope (Leica DM LFS; Leica, Wetzlar, Germany) and continuously perfused with an extracellular solution containing (in mM) 125 NaCl, 2.5 KCl, 2 CaCl_2_, 1 MgCl_2_, 1.25 NaH_2_PO_4_, 26 NaHCO_3_ and 25 glucose and aeration with 95% O_2_ and 5% CO_2_ (pH 7.4) at a rate of 2 mL/min. The organoid was held down by a weighted net to prevent it from moving. The bath temperature was maintained at 30–32°C using an in-line heater (TC-324B; Warner Instruments, Hamden, CT, United States). Whole-cell current-clamp recordings were performed using an EPC-8 patch-clamp amplifier (HEKA, Darmstadt, Germany). Patch pipettes were prepared from borosilicate glass capillaries and filled with an internal solution containing (in mM) 120 K-methylsulfate, 10 KCl, 0.2 EGTA, 2 MgATP, 0.3 NaGTP, 10 HEPES, 10 Na_2_-phosphocreatine, and 0.1 spermine, adjusted to pH 7.3 with KOH. The osmolarity of the internal solution was 280–290 mOsm/L, and the resistance of the patch electrodes was 4–8 MΩ in the bath solution. The voltage signals were low-pass filtered at 3 kHz and digitized at 10 kHz. The calculated liquid junction potential of -5 mV was corrected. The data were acquired using a pClamp 9 system (Molecular Devices, Sunnyvale, CA, United States). Voltage responses of the cells were investigated by the application of depolarizing and hyperpolarizing current pulses (400 ms in duration). Off-line analysis was performed using AxoGraph X software (AxoGraph Scientific, Berkeley, CA, United States). The input capacitance was estimated based on the current induced by a 10-mV-voltage step from a holding potential of -70 mV. The input resistance was estimated based on the voltage change induced by an applied hyperpolarizing current pulse of -40 pA. The spike amplitude was determined by the spike height from its threshold, defined as the membrane potential at which the derivative of the voltage trace reached 10 V/s. The maximum firing frequency was obtained from cells that exhibited more than one spike and calculated as the reciprocal of the shortest interspike interval between successive pairs of spikes.

### Mass Spectrometric Analysis

Human ESCs (hESCs), iPSCs, ESC-derived organoids, and iPSC-derived organoids were washed with ice-cold PBS, harvested by scraping and centrifugation, and frozen in liquid nitrogen. The frozen cells and organoids were crushed by using Multi-beads shocker (Yasui Kikai, Japan) and subsequently lysed by sonication in 9.8 M urea with protease inhibitor cocktail (cOmplete; Roche, Basel, Switzerland) and phosphatase inhibitor cocktail (PhosSTOP; Roche, Basel, Switzerland). The clear lysate was collected by centrifugation, and protein concentration was measured by BCA protein assay. Twenty micrograms of proteins was mixed with an internal standard protein mixture (10 fmol/ml MassPREP; Waters, Milford, MA, United States) and incubated with 2 mM Tri(2-carboxyethyl)phosphine hydrochloride (TCEP-HCl) for 30 min at 37°C for reduction, followed by alkylation with 55 mM iodoacetamide for 30 min at room temperature. The mixture was then diluted fourfold with 0.1 M triethylammonium bicarbonate and subjected to trypsin digestion (1:40 trypsin: sample ratio) for 3 h at 37°C. The digestion was terminated by trifluoroacetic acid, following by desalting with SDB-XC StageTips. The samples were fractionated into eight fractions by using SDB StageTips. Each fraction was dried by vacuum and dissolved in the measurement buffer (3% acetonitrile and 0.1% formic acid). Mass spectrometry was performed as described previously (Uetsuka et al., 2015). To identify the proteins, raw data of peptides were analyzed using Proteome Discoverer 2.2 (Thermo Fisher Scientific, Waltham, MA, United States) and Mascot 2.6 (Matrix Science, London, United Kingdom). The peptide results from all eight fractions were combined and subjected to search for the matching proteins in UniProt human database (The Uniprot Consortium, 2018). Maximum numbers of missed cleavages, precursor mass tolerance, and fragment mass tolerance were set to 3, 10 ppm, and 0.01 Da, respectively. The carbamidomethylation on Cys was set as a fixed modification, whereas oxidation of Met and deamidation of Asn and Gln were set as variable modifications. A filter of false discovery rate of less than 1% was applied to the data.

The Minora Feature Detector node was used for label-free quantification, and the consensus workflow included the Feature Mapper and the Precursor Ion Quantifier nodes using intensity for the precursor quantification. The protein intensities were normalized by the total peptides intensity. In addition, annotations from the Ingenuity Knowledge Base (IKB; released in autumn, 2018; Qiagen, Redwood City, CA, United States) and the database of Ingenuity Pathway Analysis were used to determine the localization and functional categories of the identified proteins.

For downstream analysis, we used the data normalized by Proteome Discoverer 2.2. All the following analysis was calculated on R. For missing value handling, we first applied listwise deletion method and removed the rows containing missing values. The removed rows are shown in Supplementary Table S5. The respective correlation coefficients between iPSCs, ESCs, iPS-derived brainstem organoid, and ES-derived brainstem organoid were calculated after log transformation, and the correlation coefficient, the distributions of all genes, and scatter plots of all the genes in each sample were shown by “pairs.panels” function in psych, R package^1^ (Supplementary Figure S1A). We performed principal components analysis (PCA) and analyzed the contribution rate of each principal component (PC) on the log-transformed data (Supplementary Figures S1B,C). Next, after trimmed mean of M values (TMM) normalization, we extracted DEGs between stem cells and brainstem organoids based on the likelihood ratio test by edgeR, R package^2^). The threshold of DEGs was *p* < 0.05.

### scRNA-Seq and Data Analysis

To dissociate hBSOs into single cells, we incubated them for ∼30 min in Accutase (Stemcell Technologies, Vancouver, British Columbia, Canada) at 37°C. Droplet-based scRNA-seq libraries were generated using the Chromium Single Cell 3’ Reagent kits V2 (10X Genomics, Pleasanton, CA, United States). Cell number and cell viability were assessed using the Countess II Automated Cell Counter (Thermo Fisher Scientific, Waltham, MA, United States). Thereafter, cells were mixed with the Single Cell Master Mix and loaded together with Single Cell 3’ Gel beads and Partitioning Oil into a Single Cell 3’ Chip. RNA transcripts were uniquely barcoded and reverse-transcribed in droplets. cDNAs were pooled and amplified according to the manufacturer’s protocol. Libraries were quantified by high-sensitivity DNA reagents (Agilent Technologies, Santa Clara, CA, United States) and the KAPA Library Quantification kit (KAPA Biosystems, Wilmington, MA, United States). Libraries were then sequenced by Illumina Hiseq 2500 in rapid mode.

Raw sequencing data from the organoid were preprocessed using the Cell Ranger (v 2.2.0; 10X Genomics, Pleasanton, CA, United States) software (Zheng et al., 2017). Reads were aligned to the GRCh38 human reference genome using STAR. After processing by Cell Ranger, the scRNA-seq data were analyzed using the Seurat v.3.0.0 R package (Satija et al., 2015). Cells with more than 8,000 or fewer than 750 detected genes, as well as cells expressing more than 5% mitochondrial genes, were excluded. Genes expressed in fewer than three cells were excluded. We collected a total of 2,345 cells expressing a total of 19,454 genes. The data sets were log normalized and scaled to 10,000 transcripts per cell. The top 2,000 highly variable genes were determined using the variance-stabilizing transformation method. The data sets were scaled and unique molecular identifier counts, ribosomal genes, and mitochondrial genes were regressed out. We analyzed the data sets by using “SCTransform” function in Surat (Hafemeister and Satija, 2019). After PCA, clustering was performed based on the top 15 PCs using the shared nearest neighbor modularity optimization with a resolution of 0.8. Cluster identities were assigned based on cluster gene markers determined by the “FindAllMarkers” function in Seurat (Supplementary Table S6).

## Results

**FIGURE 1:**
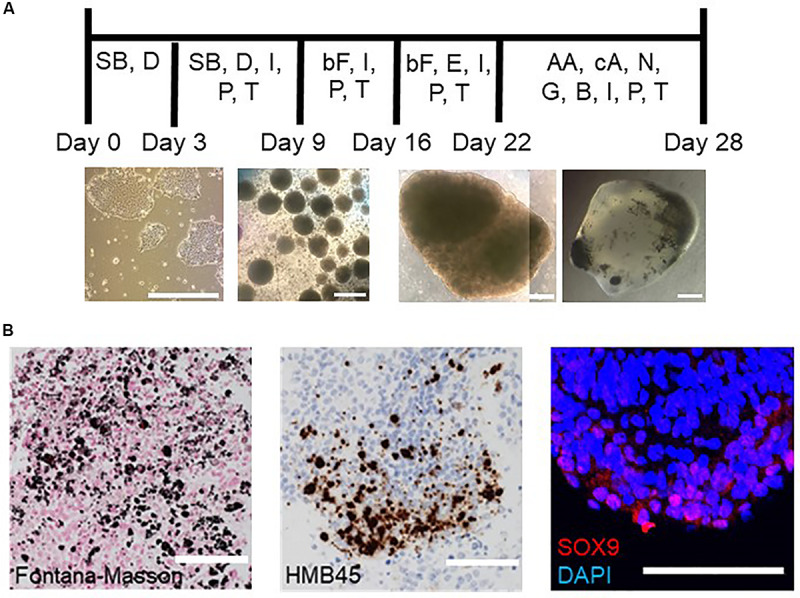
Schematic procedure of inducing hBSOs and immunohistochemical analysis of 1-month-old hBSOs from hESCs. **(A)** Schematic procedure of generating human brainstem organoids. SB, SB431542; D, dorsomorphin; I, insulin; P, progesterone; T, transferrin; bF, basic fibroblast growth factor; E, epidermal growth factor; AA, ascorbic acid; cA, cyclic adenosine monophosphate; N, neurotrophin 3; G, glial cell line–derived neurotrophic factor; B, brain-derived neurotrophic factor. Photographs of organoids were taken on days 0, 9, 22, and 28. Bars = 500 μm. **(B)** Immunohistochemistry of hBSOs at 1-month old from hESCs for the markers of melanocyte (Fontana–Masson, HMB45) and neural crest cell (SOX9). Bars = 100 μm.

To generate hBSOs from hPSCs, we used a combination of several growth factors, including EGF/bFGF for the initial proliferation of neuronal stem/progenitor cells, and BDNF, GDNF, and NT-3 for the subsequent differentiation of dopaminergic neurons and neural crest cells. This procedure is different from the protocols in previously reported studies (Figure 1A and Supplementary Figure S2). A recent study by Muotri and colleagues reported the presence of neural networks in their cortical organoids with advanced maturity (Trujillo et al., 2018). We further modified their protocol to induce dopaminergic neurons by adding insulin, transferrin, and progesterone, all of which have been shown to be protective or induce dopaminergic neuronal differentiation in two-dimensional culture (Diaz et al., 2009; Ayton et al., 2016; Lee et al., 2016). Our novel approach to generate hBSOs yielded cells with dark granules between 22 and 28 days of cultivation (Figure 1A), an observation that was absent at a similar stage in previous studies (Thomas et al., 2017; Trujillo et al., 2018).

On immunohistochemistry (IHC), we detected melanin in the hBSOs by hematoxylin–eosin, Fontana–Masson, and HMB45 stainings (Figure 1B), showing that these dark cells were melanocytes derived from neural crest cells in the organoids. The expression of *SOX9* [12.1% (74 of 608 cells)], which plays a role in the migration of neural crest cells (Spokony et al., 2002), also supported the existence of neural crest population in the brainstem organoids (Figure 1B).

**FIGURE 2:**
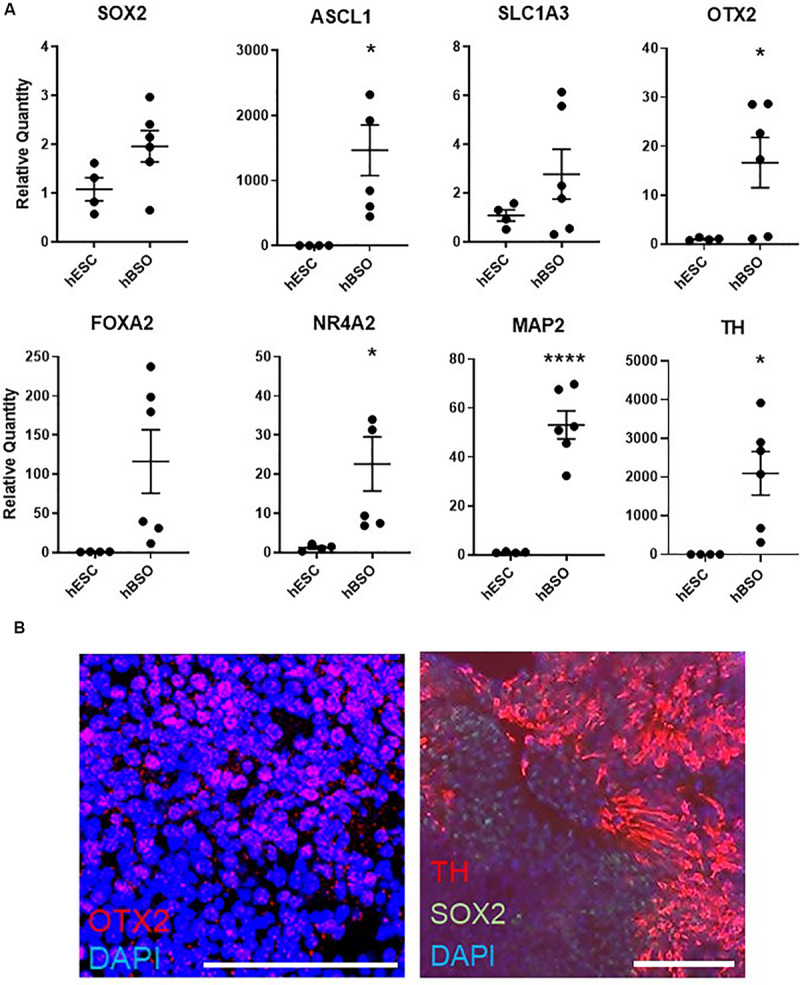
Quantitative PCR and immunohistochemical analysis of 1-month-old hBSOs from hESCs. **(A)** Quantitative PCR analysis of 1-month-old hBSOs for the markers of neural stem/progenitor cell (SOX2, Mash1, SLC1A3), mature neuron (MAP2), midbrain (OTX2), and mDA (FOXA2, NR4A2, TH). Error bars indicate mean ± SEM; *p = 0.0167 (ASCL1), *p = 0.0412 (OTX2), *p = 0.0387 (NR4A2), ****p < 0.0001 (MAP2), *p = 0.0178 (TH). **(B)** Immunohistochemical staining of midbrain marker (OTX2), mDA marker (TH), and neural stem cell marker (SOX2) at 1 month. Bars = 100 μm.

Based on a quantitative PCR (qPCR) analysis of 1-month-old hBSOs, we confirmed distinct expression of various markers for neuronal cells. Additionally, we detected the neural stem/progenitor cell markers *SOX2*, *ASCL1*, *SLC1A3*, and *OTX2* (Figure 2A), which are necessary for the development of anterior brain structures including the midbrain (Wurst and Prakash, 2014). Our analyses also demonstrated the expression of *FOXA2*, a potent inducer of midbrain dopaminergic (mDA) progenitors (Sasaki and Hogan, 1993; Norton et al., 2005; Kittappa et al., 2007; Lin et al., 2009; Ribes et al., 2010), and *NR4A2*, which is essential for both the survival and final differentiation of ventral mesencephalic late dopaminergic precursor neurons into dopaminergic neurons (Saucedo-Cardenas et al., 1998), and *SOX6*, important for the specification of substantia nigra dopamine neurons (Panman et al., 2014; Figure 2A and Supplementary Figure S3). We also detected the expression of *LMX1A*, required to trigger dopamine cell differentiation (Andersson et al., 2006), and *EN1*, required in early development of mDA neuron (Alves dos Santos and Smidt, 2011), in hBSOs, and both genes were not revealed in hESCs (Supplementary Table S3). Consistently, we detected the expression of mRNAs coding for the pan-neuronal marker *MAP2* and the mature dopaminergic neuronal marker *TH* (Figure 2A). Using IHC, we demonstrated that the organoids have midbrain components via the detection of protein expressions of SOX2 [26.8% (153 of 571 cells)], OTX2 [16.5% (55 of 333 cells)], and TH [14.7% (84 of 571 cells)], which also indicated that the organoids contain midbrain components (Figure 2B). Other midbrain or mDA markers were also detected in 1-month hBSOs by qPCR (Supplementary Figure S3 and Supplementary Table S3).

**FIGURE 3:**
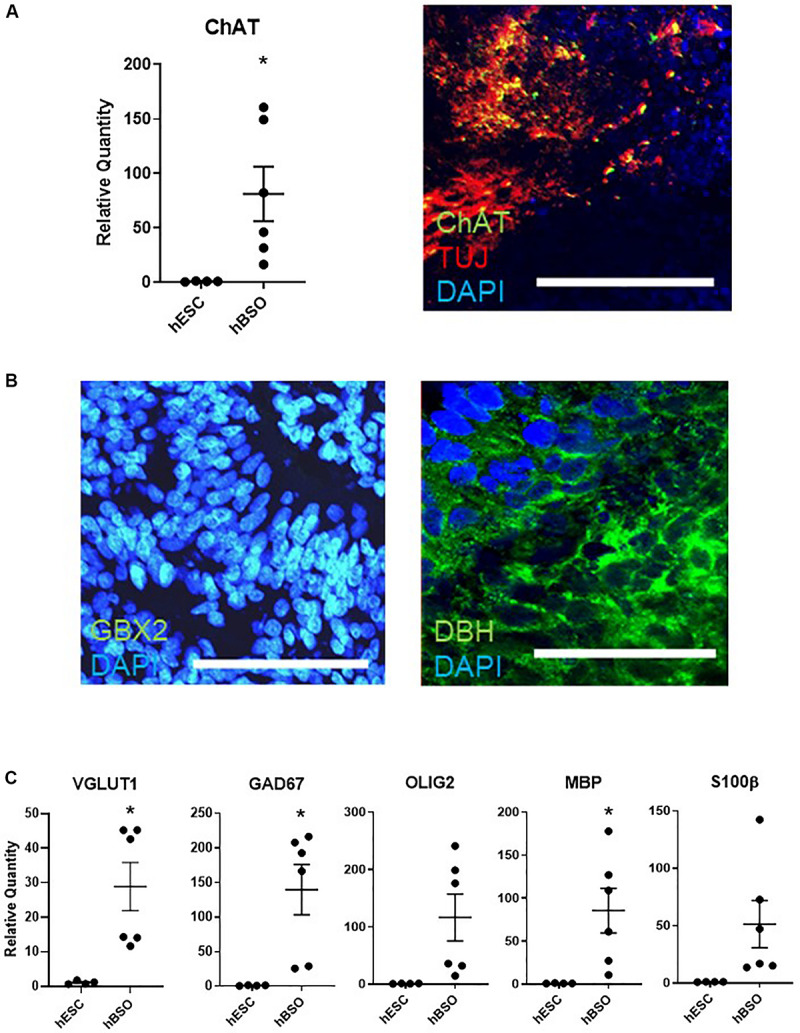
Quantitative PCR and immunohistochemical analysis of 1-month old hBSOs from hESCs. **(A)** Quantitative PCR analysis and immunohistochemical staining of the marker of cholinergic neuron (ChAT). Error bars indicate mean ± SEM; *p = 0.0337 (ChAT). Bars = 100 μm. **(B)** Immunohistochemical staining of DBH, the marker of noradrenergic neuron, and hindbrain marker (GBX2). Bars = 100 μm. **(C)** Quantitative PCR analysis for the marker of excitatory neuron (VGLUT1), inhibitory neuron (GAD67), oligodendrocyte (OLIG2, MBP), and astrocyte (S100β). Error bars indicate mean ± SEM; *p = 0.0127 (VGLUT1), *p = 0.0155 (GAD67), *p = 0.0320 (MBP).

Furthermore, we observed expressions of *ChAT* on qPCR and IHC [17.4% (83 of 478 cells)] (Figure 3A). The detection of ChAT, a marker for cholinergic neurons, suggests the existence of medulla population (Stornetta et al., 2013). GBX2 is a hindbrain marker that plays a role in the positioning of the midbrain/hindbrain boundary with OTX2. Its expression [in 15.2% (44 of 289) cells] suggests that the hBSOs included midbrain and hindbrain population (Waters and Lewandoski, 2006; Figure 3B). The expression of DBH [13.9% (63 of 453 cells)], a marker for the central noradrenergic nervous system, may indicate that pons and medulla components are contained in the hBSOs (Swanson and Hartman, 1975; Figure 3B). Also, we detected *VGLUT1* and *GAD67*, markers for mature and functional excitatory and inhibitory neurons, respectively (Soghomonian and Martin, 1998; Fremeau et al., 2004). The expression of *OLIG2* and *MBP* indicated that our organoids contained oligodendrocyte progenitors and mature oligodendrocytes (Wei et al., 2003), whereas the presence of S100β suggested the existence of astrocytes (Figure 3C and Supplementary Figure S3). In addition, the qPCR analysis on 3-month-old hBSOs demonstrated the expressions of a variety of neuronal components (Supplementary Figure S4 and Supplementary Table S4).

To further verify the translated products in hBSOs, we performed protein mass spectrometric analysis of the hBSOs at 1 month. Finally, we identified 3,458 DEGs, of which 763 genes satisfied false recovery rate (FDR) <0.05. Of these 763 proteins, we identified genes found to be enriched in brainstem, cerebellum, or basal ganglia (Table 1), suggesting that hBSOs have specific components for brainstem or cerebellum (Uhlen et al., 2015, 2017; Thul et al., 2017).

**TABLE 1:** Differentially expressed genes specific for brain in mass spectrometric analysis (FDR < 0.05).

Regional specificity	Symbol	Accession	logFC	logCPM	LR	*P* value	FDR
Pons and medulla	DSG2	Q14126	5.669599673	6.550998938	58.16620526	2.41E-14	7.57E-12
	PRPH	P41219	−6.447981469	6.171664268	44.05998978	3.18E-11	3.84E-09
	KRT8	P05787	3.759699355	10.38106493	21.58664839	0.00000338	0.0000829
	KRT18	P05783	3.725473938	10.34020675	23.87534344	0.00000103	0.000032
	PRSS8	Q16651	2.094239437	−0.279713416	8.491513239	0.003568069	0.02138368
	CFAP44	Q96MT7	−2.431897956	2.251822919	9.751540469	0.001791725	0.012801209
Cerebellum	JARID2	Q92833	8.121840088	2.982965594	69.44954272	7.84E-17	5.42E-14
	HIST3H2BB	Q8N257	−4.40083852	1.304593593	19.49310574	0.0000101	0.00020181
	HELLS	Q9NRZ9	2.112404374	6.517273007	12.01916385	0.000526563	0.004961459
	ZIC2	O95409	1.958589485	3.966265294	7.992220317	0.004697877	0.026045008
Cerebellum, midbrain, pons, medulla	MAB21L1	Q13394	−3.26983685	1.866012194	13.63006394	0.000222592	0.002412928
Basal ganglia	PCP4	P48539	−2.607799879	3.661385349	10.15879101	0.001436148	0.010890787

**FIGURE 4:**
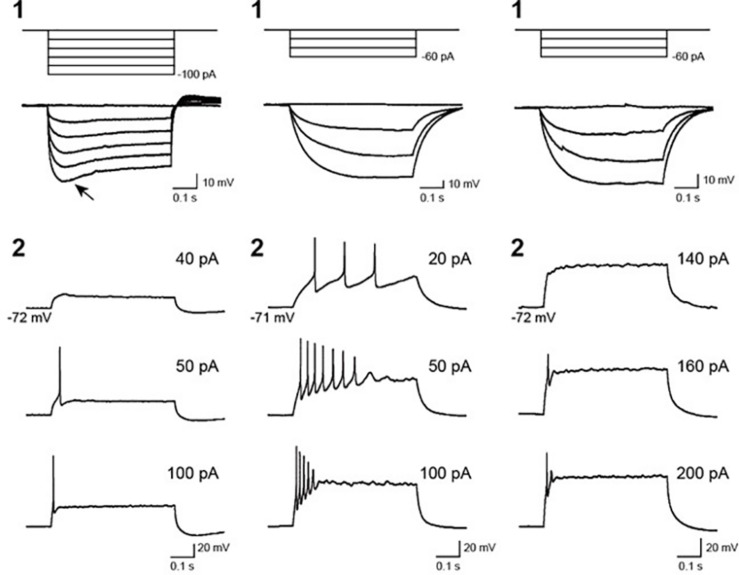
Voltage responses of recorded cells in 1-month-old hBSOs from hESCs to current pulses. (Left, middle, right) Three types of cells exhibiting different hyperpolarizing and firing responses. (1) Voltage responses to hyperpolarizing current pulses. Arrow: voltage responses characterized by a voltage sag. (2) Firing responses to depolarizing current pulses. Firing responses with multiple spikes (left), immature spikes (middle), and a few spikes (right). The values of depolarizing current pulses are given at *right*.

To assess the electrical functionality of the neurons in the hBSOs, we performed electrophysiological characterization using the whole-cell patch clump method. Most cells displayed neither an action potential nor a membrane potential less than -40 mV immediately after patch membrane rupture. One cell showed hyperpolarizing voltage responses with an obvious voltage sag, defined as a fast hyperpolarization, followed by a slow depolarization (Figure 4, left-1, arrow), whereas other cells (*n* = 8) showed no sag (Figure 4, middle-1, right-1). In the neurons exhibiting repetitive firings, the spike overshot (52.9 ± 5.5 mV in amplitude) and its width was narrow (1.1 ± 0.3 ms of the half width). However, in the neuron exhibiting a few spikes, the spike amplitude was small (33.9 ± 7.9 mV), and the half width was wide (2.8 ± 1.6 ms), suggesting that these neurons were still in the course of development.

To further analyze the gene expression profile of hBSOs, we carried out total RNA sequencing (RNA-seq) analysis of hCOs induced using Lancaster and colleagues’ protocol (Lancaster et al., 2013; Lancaster and Knoblich, 2014) and the hBSOs at day 28. RNA-seq analysis revealed that the hBSOs contained cell populations like that of a human brainstem. At the age of 1 month, the hBSOs expressed genes that were characteristic of a fetal midbrain, such as *LMX1A* and *LMX1B*, and those indicating dopaminergic neuronal property, such as *EN1*, *EN2*, *TYR*, and *TH*, whose expression was stronger than in hCOs (Supplementary Table S7). Additionally, *MLANA* and *MITF*, known as melanocyte-marker genes, and *MBP*, a marker for oligodendrocytes, also showed higher expressions in the hBSOs. We also observed significant expression of NGF and SOX9 specific to neural crest–stem cells. On the other hand, cortical neuron specific markers, such as Reelin and Lhx2, were lower in the hBSOs than the hCOs, indicating their distinct cellular populations.

**FIGURE 5:**
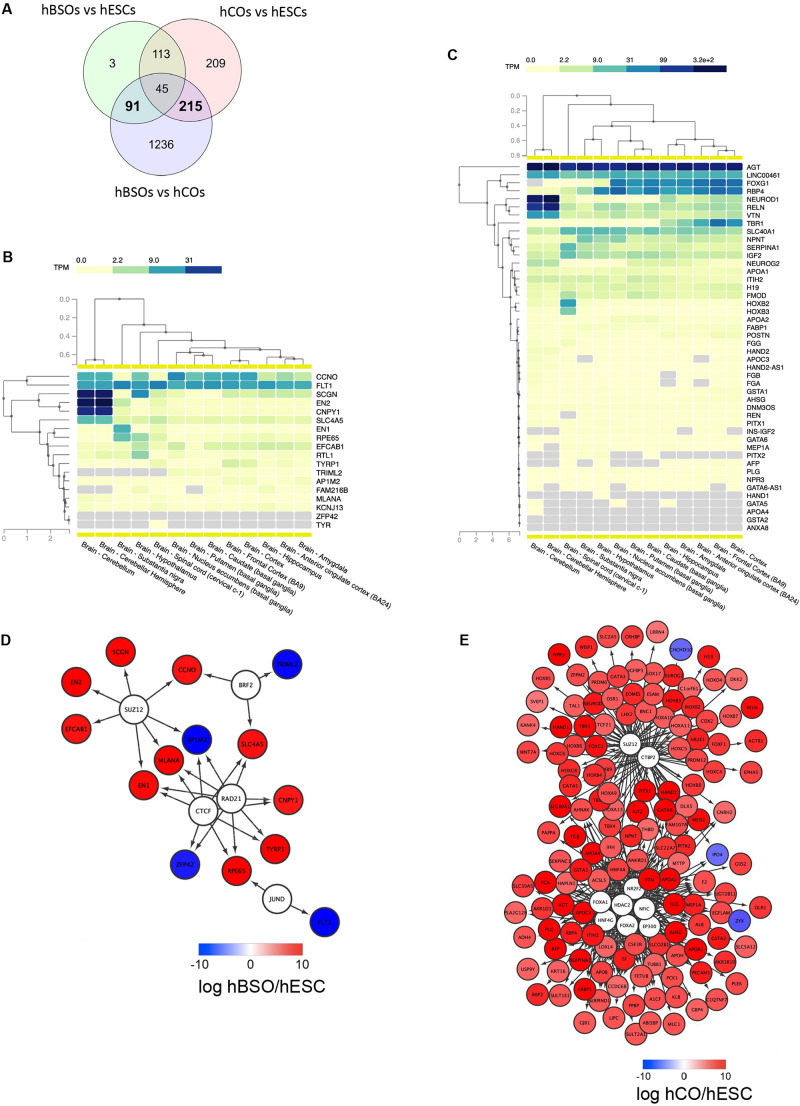
RNA-seq transcriptomic analysis of 1-month old hBSOs from hESCs. **(A)** Venn diagrams of the number of genes differentially expressed between human brainstem organoids (hBSOs), human cerebral organoids (hCOs), and hESCs. **(B)** Clustering brain regions based on the expression of differentially expressed genes selective in hBSOs (FDR < 10%). **(C)** Clustering brain regions based on the expression of differentially expressed genes selective in hCOs (FDR < 3%). **(D)** Transcription factors that potentially regulate the differentially expressed genes in hBSOs. **(E)** Transcription factors that potentially regulate the differentially expressed genes in hCOs.

To better understand the molecular mechanism regulating the differentiation of the hBSOs, we identified DEGs (Figure 5A) between the hBSOs and hESCs (Supplementary Table S8) and between the hCOs and hESCs (Supplementary Table S9). We detected 91 DEGs that were selectively regulated in the hBSOs (Supplementary Table S10) and 215 DEGs in the hCOs (Supplementary Table S11).

To analyze the correlation between the genes selectively regulated in the hBSOs, the hCOs, and various parts of the brain, we used GTEx, a comprehensive public resource to study tissue-specific gene expression and regulation (Figures 5B,C) (GTEx Consortium, 2013). High expressions of *EN2*, *CNPY1* reflected the link between the hBSOs, human cerebellum, and substantia nigra, whereas the expression of *EN1* and *RPE65* demonstrated the relationship between the hBSOs, substantia nigra, and hypothalamus (Figure 5B). Low expression of *TRIML2* in the hBSOs is characteristic of the cerebellum, substantia nigra, and hypothalamus (Figure 5B).

To identify TFs potentially governing the genes selectively regulated in the hBSOs and the hCOs, we applied iRegulon (Janky et al., 2014), a computational method built upon the fact that genes coregulated by the same TF contain common TF-binding sites and that uses the gene sets derived from ENCODE ChIP-seq data (Gerstein et al., 2012). *CTCF*, *RAD21*, *BRF2*, *JUND*, and *SUZ12* were identified as potential TFs for genes selectively regulated in the hBSOs (Figure 5D). This suggested that *EN1* and *CNPY1*, related to dopaminergic neurons, were controlled by *CTCF* and *RAD21*. Also, MLANA, one of the melanocyte markers, was indicated to be controlled by *CTCF*, *RAD21*, and *SUZ12*. On the other hand, in the hCOs, *FOXA1*, *FOXA2*, *HDAC2*, *EP300*, *NFIC*, *HNF4G*, *NR2F2*, *CTBP2*, and *SUZ12* were detected as potential TFs, and more complex and wide variety of factors were shown (Figure 5E).

**FIGURE 6:**
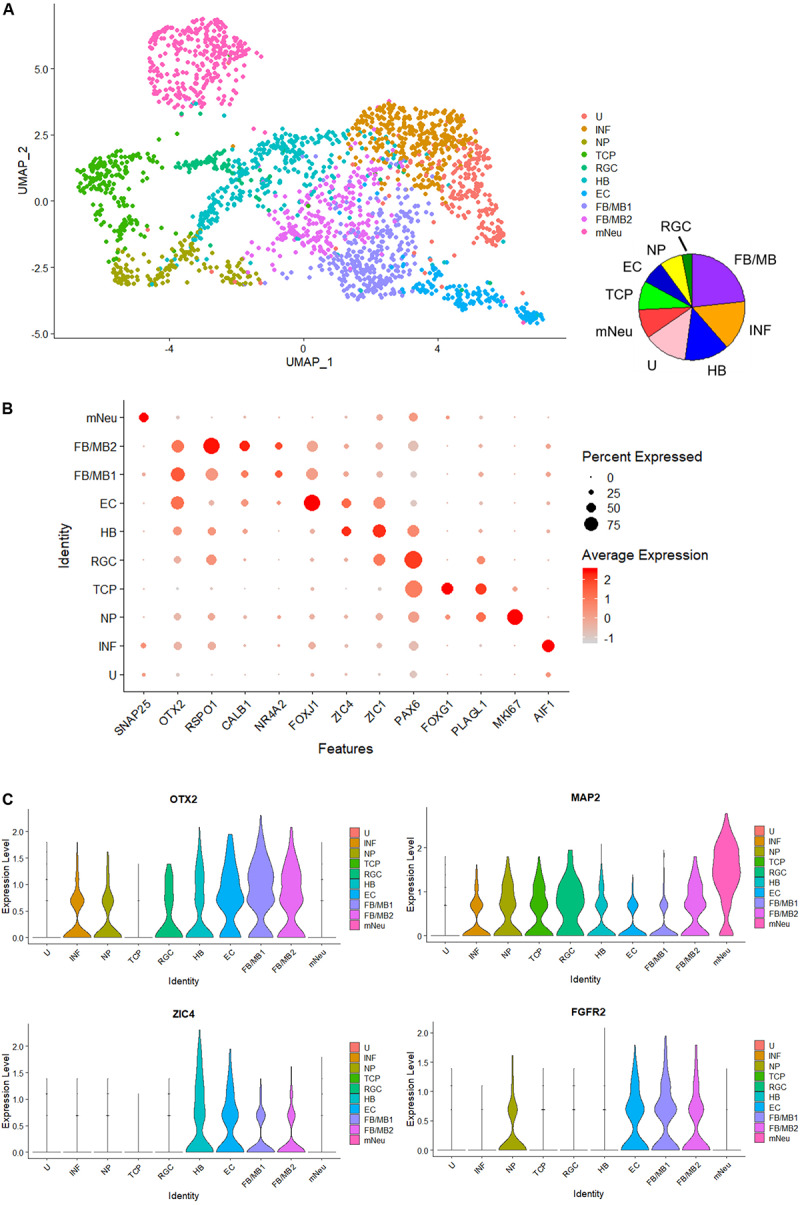
Single-cell RNA-seq analysis of 1-month-old hBSOs from hESCs. **(A)** Unsupervised clustering of all cells from human brainstem organoids. INF; inflammation, NP; neuronal progenitors, TCP; telencephalic progenitors, RGC; radial glia cells, HB; hindbrain, EC; ependymal cells, FB/MB; forebrain and midbrain, mNeu; mature neurons, U; unknown. **(B)** Dot plots showing a selection of genes that identify cell population types. **(C)** Cell distribution plot of OTX2, FGFR2, ZIC4, and MAP2.

Finally, to investigate heterogeneity and gene expression dynamics in hBSOs, we performed scRNA-seq analysis on 1-month-old hBSOs. After processing, quality control, and filtering, we analyzed a total of 2,345 cells expressing 19,454 genes. To identify distinct cell populations based on shared and unique patterns of gene expression, we performed dimensionality reduction and unsupervised cell clustering using uniform manifold approximation and projection (UMAP) (Figure 6A). The UMAP plot revealed 10 distinct cell populations composed of various cell types. Cell populations were identified based on cluster gene markers (Supplementary Table S6) and the expression of known marker genes. We could not annotate cluster 1 and termed this cluster as “unknown” (U). Dot plot showed a selection of genes that can be used to identify cell population types (Figure 6B). Each cell population expressed canonical cell type markers. Violin plots showed the expression intensity distribution of the marker genes in each cluster (Figure 6C and Supplementary Figure S5). The neuronal progenitors cluster expressed genes of cell proliferation (e.g., *MKI67*) and neural stem cell markers (e.g., *PLAGL1*). The radial glia cells cluster expressed PAX6 and the telencephalic progenitors cluster expressed genes related to telencephalon development (*FOXG1*, *LHX2*) (Godbole et al., 2018). The ependymal cells cluster expressed genes related to cilia development and formation (*FOXJ1*, *PIFO*) (Jacquet et al., 2009). The forebrain and midbrain (FB/MB) clusters expressed genes of forebrain and midbrain progenitors (*OTX2*). In addition, the FB/MB clusters expressed dopaminergic (*FGFR2*, *NR4A2*, *LMX1A*, *CALB1*), serotonergic (*HTR2C*), and melanocyte development and differentiation (MITF) markers (Quilter et al., 2012; Ratzka et al., 2012; Anderegg et al., 2015). The mature neurons (mNeu) cluster expressed pan-neuronal (*MAP2*, *SNAP25*), cholinergic (*ACHE*), and glutamatergic (*SLC17A6*) markers. The hindbrain cluster expressed genes of cerebellar or medulla formation (*ZIC1*, *ZIC4*) (Blank et al., 2011). The inflammation cluster expressed genes of microglia cells (*AIF1*) and endothelial cells (*ICAM1*). The scRNA-seq indicated that the organoids contain various cell types and neuronal subtypes.

## Discussion

To the best of our knowledge, this is the first time hBSOs with dark cells such as melanocytes have been successfully induced. These cells are of neural crest origin and derived from the fetal midbrain–hindbrain boundary. Using qPCR, IHC, RNA-seq, scRNA-seq, and mass spectrometry, we observed a gene expression profile like that of a human fetal brainstem in hBSOs that were cultivated for 28 days. We built our current protocol upon existing protocols for human brain organoids. In particular, we were inspired by Lancaster and colleagues (Lancaster et al., 2013; Lancaster and Knoblich, 2014), who found that their organoids, acquired with the least use of growth factors, were composed of neuroectodermal tissues with multiple identities, such as cerebral cortex, hippocampus, and retina. In another key study, Muotri and colleagues reported a protocol where cortical organoids were induced with electrophysiologically active neurons with glutamatergic and GABAergic signaling (Trujillo et al., 2018). We designed our protocol based on these existing reports and made use of selected hormones, such as human insulin, transferrin, and progesterone, which have been found to be protective or support the survival and differentiation of dopaminergic neurons and neural crest lineage cells (Ciarlo et al., 2017; Frith and Tsakiridis, 2019). Of note, our findings suggest that the use of an orbit shaker might have contributed to quick induction of hBSOs, through the achievement of an ideal concentration gradient of growth factors. Considering that our protocol does not contain sonic hedgehog (SHH) or WNT, essential for inducing ventral midbrain (Tang et al., 2010), and that the presence of melanocytes implies the presence of dorsal tissues of brainstem, the evidence of mDA neurons in hBSOs is interesting. It is plausible that these factors were secreted from neighboring cells in the population of hBSOs.

The hBSOs and previously reported midbrain organoids contained characteristic dark spots mimicking the existence of neuromelanin. Given that neuromelanin is derived over a few years, through an accumulation of catecholamine-derived wastes, the dark spots observed in previously reported midbrain–organoid protocols may have melanocytes such as those found in the hBSOs. However, to our knowledge, no existing study on midbrain organoids has identified such neural crest–derived cells in their organoids.

Our findings indicate that the hBSOs mock broad fetal brainstem region and surrounding neural crest, as cranial melanoblasts are known to originate from the neural crest around the midbrain and spread to the whole cranial region (Adameyko et al., 2012; Denecker et al., 2014). Our observations of the high Wnt1 expressions in the hBSOs, which characterize the neural crest (Adameyko et al., 2012), are consistent with this idea. Our current findings also provide the basis for future research on hereditary diseases caused by neural crest migration disorders.

We would like to propose two potential applications based on our current findings: (1) to use hBSOs as a tool for drug screening and (2) apply hBSOs as an efficient tool for the modeling of neural crest disorders. First, the quick induction of hBSOs will enable more efficient drug screenings and accelerated research on the molecular mechanisms driving brainstem neurodegenerative diseases. Electrophysiological analysis of the hBSOs at the age of 1.5 months revealed neurons that exhibited action potentials and hyperpolarizing responses with voltage sags attributed to the activation of hyperpolarization-activated cyclic nucleotide-gated (HCN) cation channels (Robinson and Siegelbaum, 2003; He et al., 2014). This finding suggests that HCN channels, as well as spike generating Na^+^ and K^+^ channels, are expressed at an early stage of the hBSO. It also suggests the presence of a heterogeneous neuronal population that is capable of exhibiting distinct electrophysiological properties in the organoid.

A second potential application of hBSOs lies in their utility in investigations into the interaction between the brainstem and neural crest cells. For example, brainstem functions are reported to be affected in representative neural crest disorders, DiGeorge syndrome, and Waardenburg-Shah syndrome (Wang et al., 2017; Nusrat et al., 2018). Nonetheless, the disease models of such diseases have yet to be established, and their pathologies remain to be known. We see the potential in applying the hBSOs developed in our current study as they contain neural crest cells and can be powerful tools for elucidating the mechanisms driving such neural crest diseases.

## Data Availability Statement

The data sets generated and analyzed during the current study are available under following accession numbers DRA009864 (RNA-seq) in DDBJ (DNA Data Bank of Japan^3^), JPST000707 (mass spectrometry) in JPOST (Japan Proteome Standard Repository/Database^4^), and GSE145306 (scRNA-seq) in GEO (Gene Expression Omnibus^5^).

## Author Contributions

NE, TM, JL, JS, KS, and EM designed the study. NE, TM, JL, MMatsubayashi, HN, TShiota, NI, TKiriyama, CZ, TKouno, YL, PK, PW, YMS, RN, TKomeda, NM, FK, MJ, SK, MN, MH, YS, TShiromizu, YN, TKasai, MT, HKobayashi, YI, YT, MMakinodan, TKishimoto, HKuniyasu, SN, JS, KS, and EM conducted the research. NE, TM, JL, YS, TShiromizu, YN, TKasai, MT, SN, AM, JS, KS, and EM analyzed the data. NE, TM, JL, KK, YS, TShiromizu, YN, TKasai, MT, SN, JS, KS, and EM wrote the manuscript. All authors contributed to manuscript revision, read and approved the submitted version.

## Conflict of Interest

The authors declare that the research was conducted in the absence of any commercial or financial relationships that could be construed as a potential conflict of interest.

## Footnotes

1 https://cran.r-project.org/web/packages/psych/index.html

2 https://bioconductor.org/packages/release/bioc/html/edgeR.html

3 https://ddbj.nig.ac.jp/DRASearch/

4 https://repository.jpostdb.org/

5 https://www.ncbi.nlm.nih.gov/geo/
