# Supplementary material for: Brainstem Organoids From Human Pluripotent Stem Cells
Source: Front Neurosci. 2020 Jun 26;14:538. doi: 10.3389/fnins.2020.00538 (PMC7332712; doi:10.3389/fnins.2020.00538)
Supplement: Supplementary file 1 [file Image_1.PDF]

Supplemental Information

Supplemental Figures and Legends

Figure S1

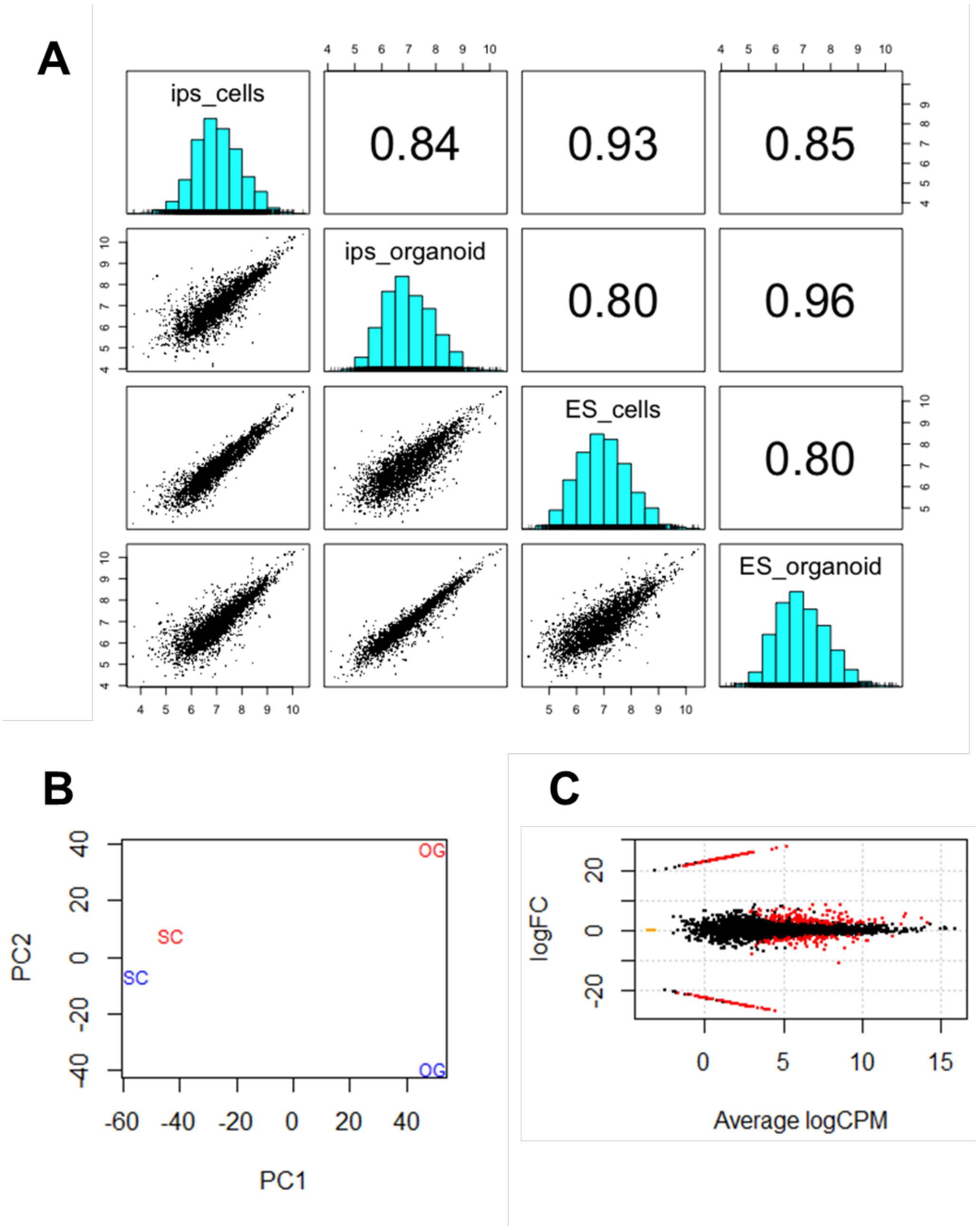

Figure S1: Mass spectrometric analysis of 1-month old hBSOs. Related to Table 1.

(A) The correlation coefficient between hiPSC, hESC, hiPSC-derived BSO and hESC-derived BSO. The distributions of all genes and scatter plots of all genes in each sample are shown. (B) Principal Component analysis of hPSCs and human brain organoids. red: iPS cell derived, blue: hEScell derived, SC: stem cell, OG: organoids. (C)MA plot for differential expression analysis (FDR<0.02).

Figure S2

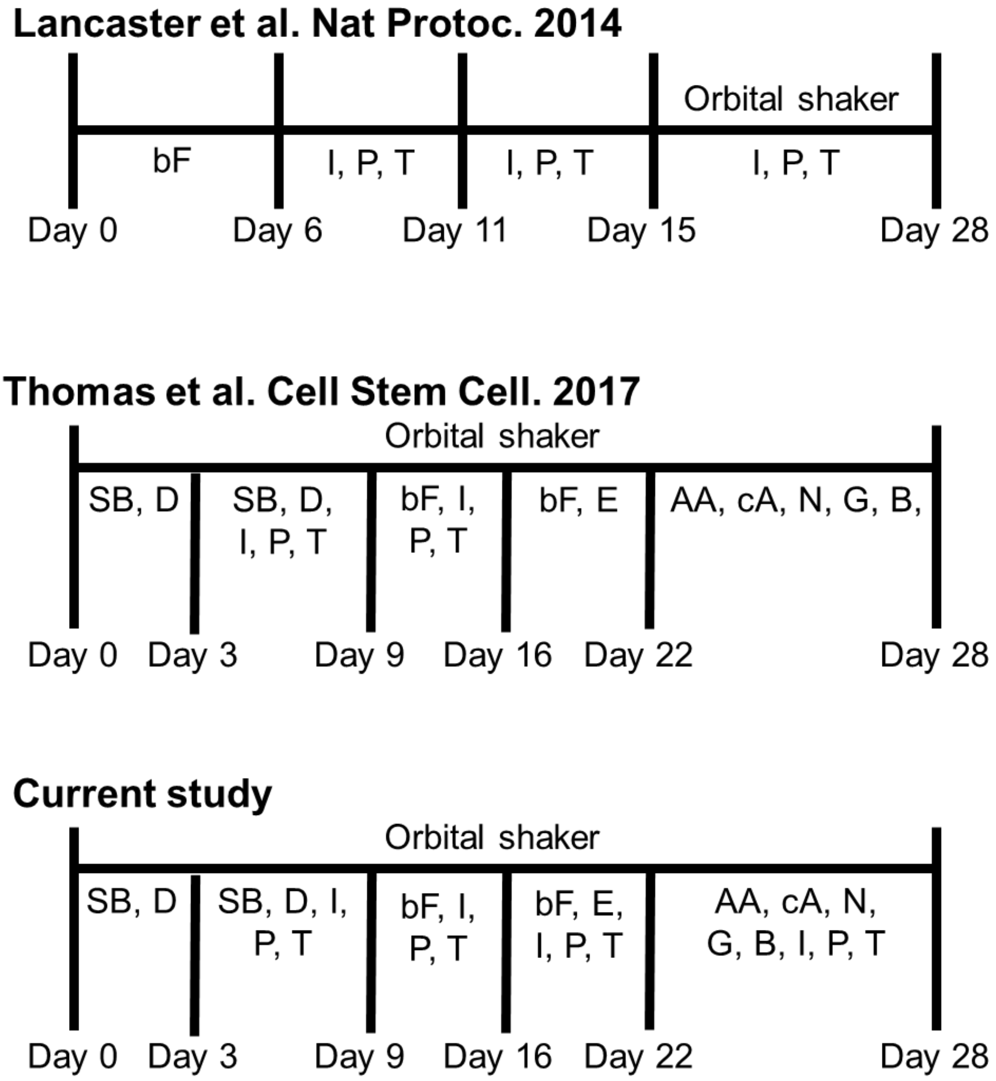

Figure S2: Schematic procedures of brain organoid by different groups. Related to Figure 1.
Procedures of organoid generation by three different groups, Lancaster et al., Thomas et al., and current study. SB, SB431542; D, dorsomorphin; I, insulin; P, progesterone; T, transferrin; bF, basic fibroblast growth factor; E, epidermal growth factor; AA, ascorbic acid; cA, cyclic adenosine monophosphate; N, neurotrophin 3; G, glial cell line derived neurotrophic factor; B, brain derived neurotrophic factor.

Figure S3

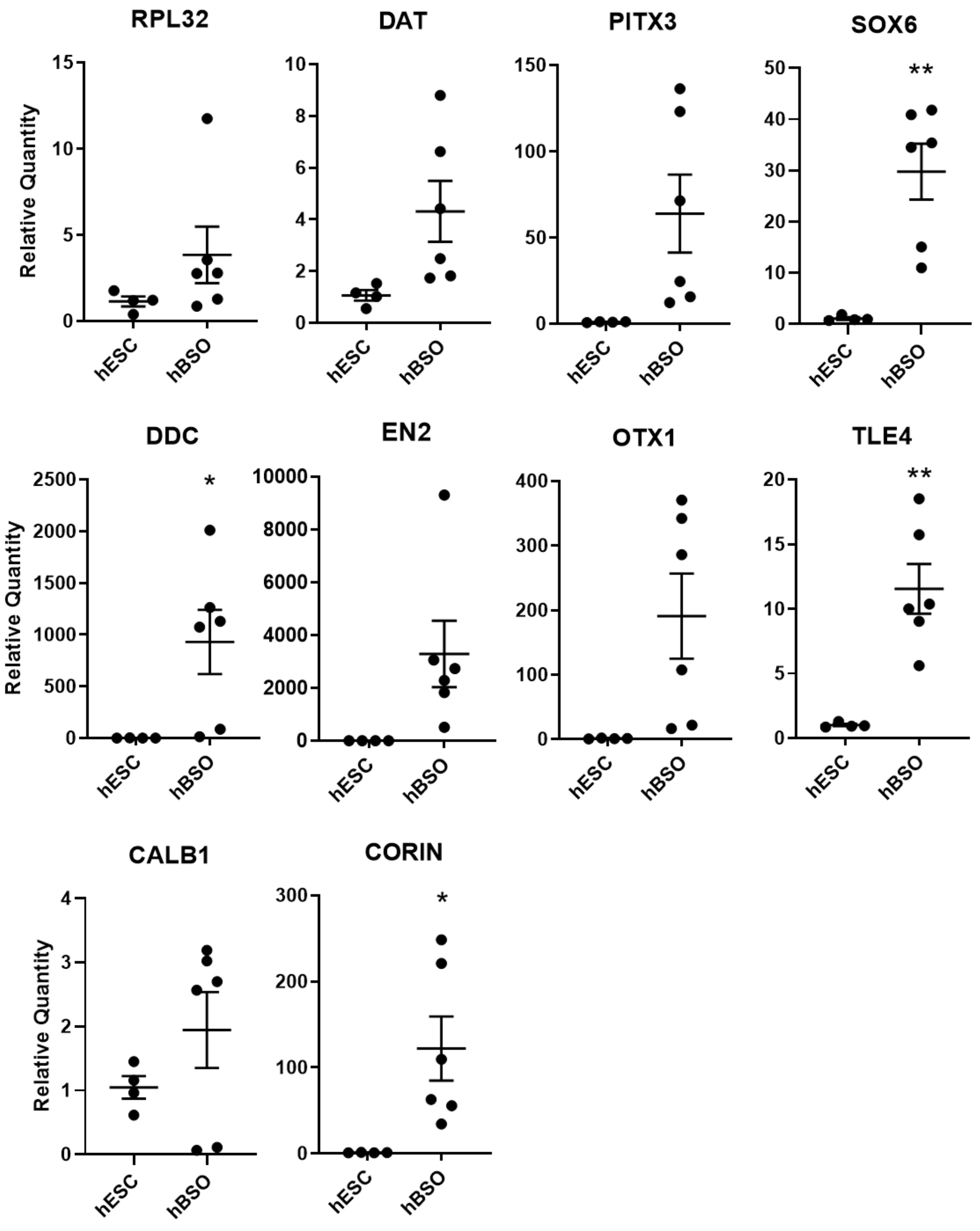

Figure S3: qPCR analysis for midbrain or mDA markers of hBSOs at one month from hESCs. Related to Figures 2 and 3.

qPCR analysis for the marker of midbrain or dopaminergic (RPL32, DAT, LMX1b, PITX3, EN1, EN2, DDC, OTX1, TLE4, CALB1, CORIN, GIRK2). Error bars indicate mean ± SEM; **p = 0.004 (LMX1b), *p = 0.0138 (EN1), *p = 0.0438 (DDC), **p = 0.0024 (TLE4), *p = 0.0316 (CORIN).

Figure S4

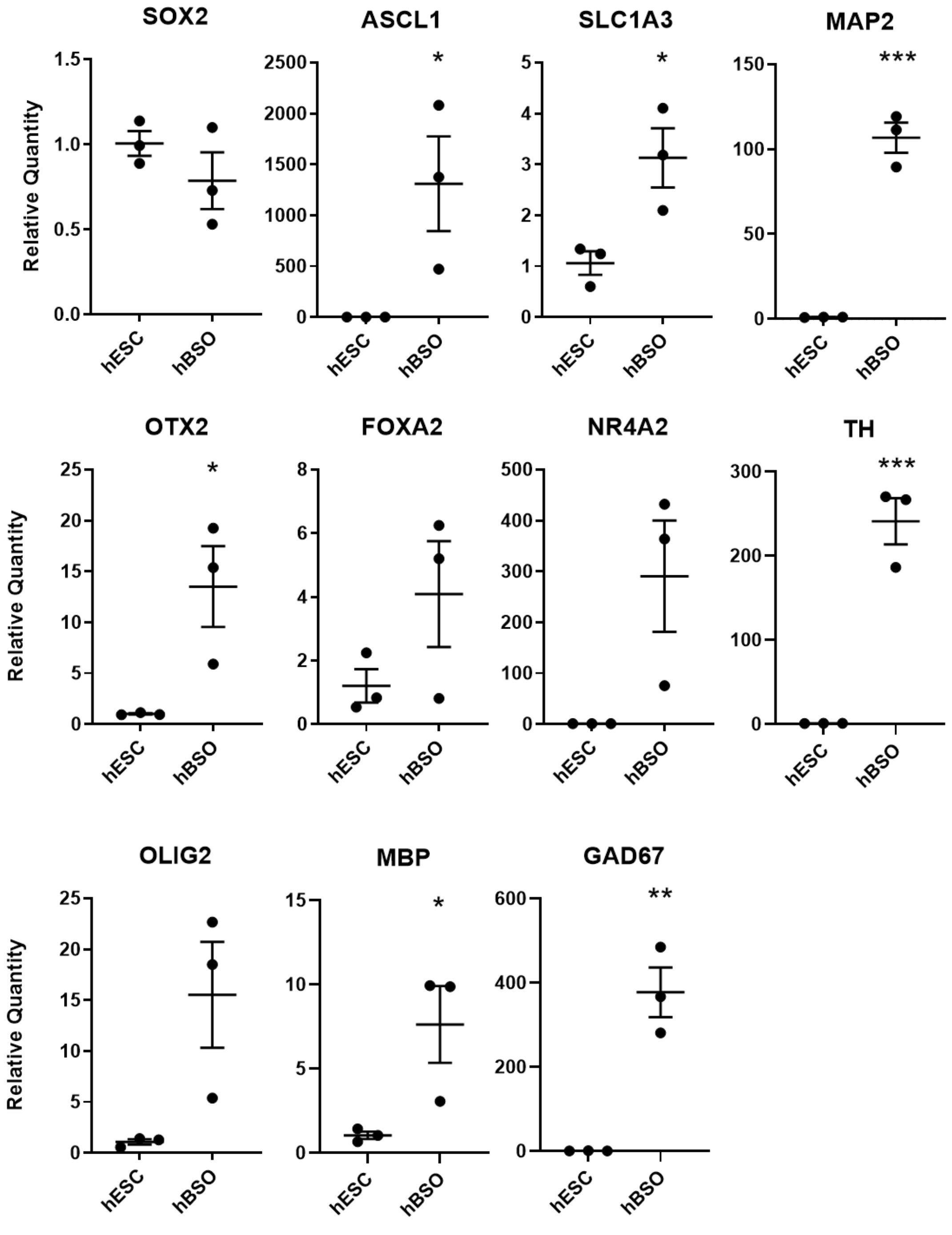

Figure S4: qPCR analysis of hBSOs at three months old from hESCs. Related to Figures 1 and 2.

qPCR analysis of 3-month old human brainstem organoids for the markers of neural stem/progenitor cell (SOX2, ASCL1, SLC1A3), mature neuron (MAP2), midbrain (OTX2) and mDA (FOXA2, NR4A2, TH), oligodendrocyte (OLIG2, MBP), inhibitory neuron (GAD67). Error bars indicate mean ± SEM; *p = 0.0298 (SLC1A3), ***p = 0.0003 (MAP2), *p = 0.0343 (OTX2), ***p = 0.0009 (TH), *p = 0.0453 (MBP), **p = 0.0031 (GAD67).

Figure S5

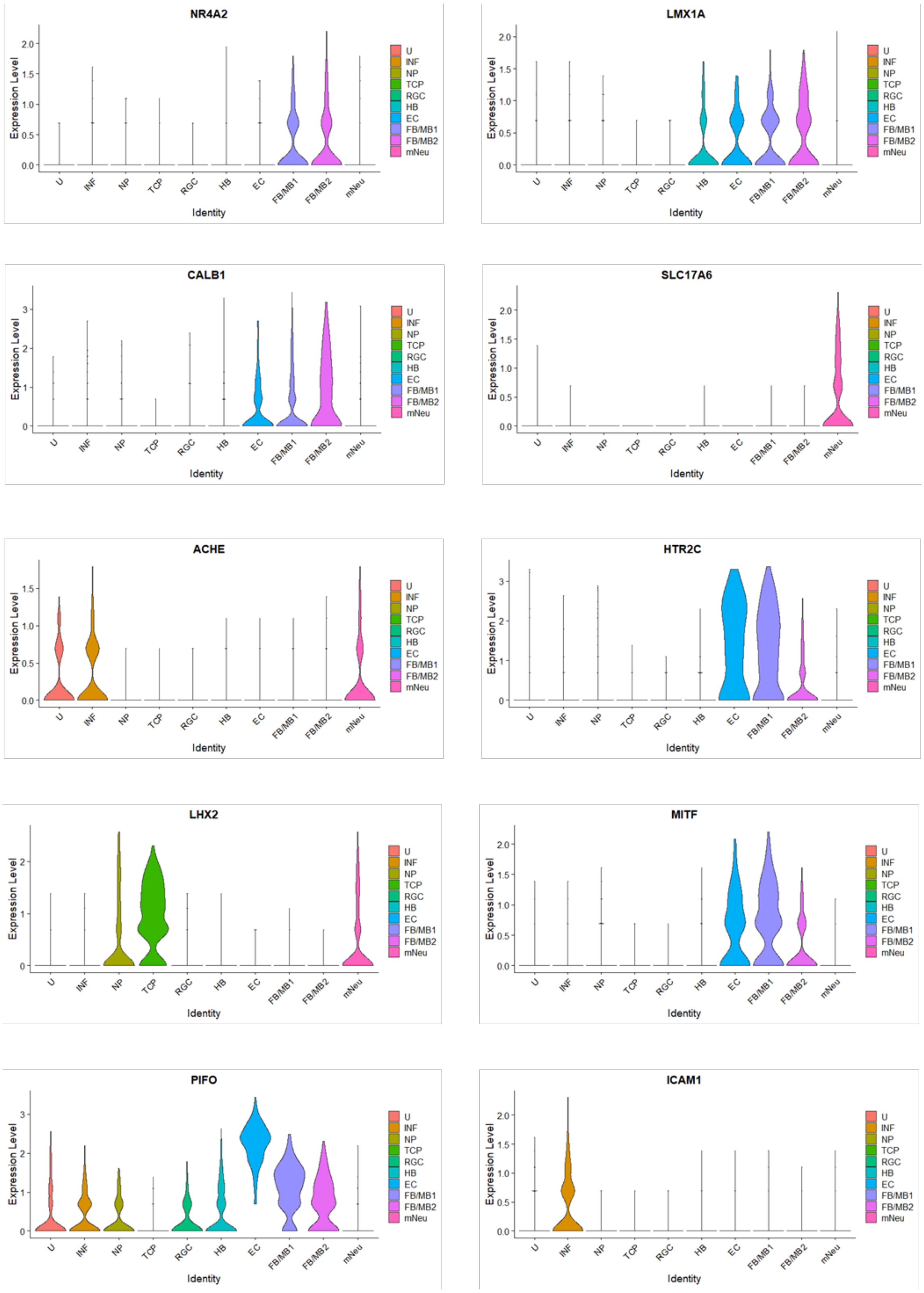

Figure S5: scRNA-seq analysis of 1-month old hBSOs from hESCs. Related to Figure 6.

Cell distribution plot of NR4A2, LMX1A, CALB1, SLC17A6, ACHE, HTR2C, LHX2, MITF, PIFO, ICAM1.
